# A Single Bacterium Capable of Oxidation and Reduction of Iron at Circumneutral pH

**DOI:** 10.1128/spectrum.00161-21

**Published:** 2021-08-25

**Authors:** Shingo Kato, Moriya Ohkuma

**Affiliations:** a Japan Collection of Microorganisms, RIKEN BioResource Research Center, Tsukuba, Ibaraki, Japan; University of Minnesota

**Keywords:** *Rhodoferax*, chemolithotrophy, iron cycling, iron oxidizers, iron reduction

## Abstract

Fe(II)-oxidizing microorganisms and Fe(III)-reducing microorganisms, which drive the biogeochemical Fe cycle on the Earth’s surface, are phylogenetically and ecologically diverse. However, no single organism capable of aerobic Fe(II) oxidation and anaerobic Fe(III) reduction at circumneutral pH have been reported so far. Here, we report a novel neutrophilic Fe(II)-oxidizing *Rhodoferax* bacterium, strain MIZ03, isolated from an iron-rich wetland in Japan. Our cultivation experiments demonstrate that MIZ03 represents a much more versatile metabolism for energy acquisition than previously recognized in the genus *Rhodoferax*. MIZ03 can grow chemolithoautotrophically at circumneutral pH by oxidation of Fe(II), H_2_, or thiosulfate as the sole electron donor under (micro)aerobic conditions (i.e., using O_2_ as the sole electron acceptor). In addition, it can reduce Fe(III) or nitrate under anaerobic conditions. Thus, this is the first report demonstrating the presence of a single bacterium capable of both Fe(II) oxidation and Fe(III) reduction at circumneutral pH. The observed physiology was consistent with its 4.9-Mbp complete genome encoding key genes for iron oxidation/reduction (*foxEY* and *mtrABC*), for nitrate reduction (*narGHI*), for thiosulfate oxidation (*soxABCDXYZ*), and for carbon fixation via the Calvin cycle. Our metagenomic survey suggests that there are more *Rhodoferax* members capable of Fe(II) oxidation and Fe(III) reduction. Such bifunctional *Rhodoferax* may have an ecological advantage in suboxic/anoxic environments at circumneutral pH by recycling of Fe as the electron donor and acceptor.

**IMPORTANCE** The biogeochemical cycle of iron (Fe) via reactions of oxidation, reduction, precipitation, and dissolution is involved in the cycle of other ecologically relevant elements, such as C, N, P, S, As, Co, Ni, and Pb. The Fe cycle on the Earth’s surface is driven by a variety of Fe(II)-oxidizing microorganisms and Fe(III)-reducing microorganisms. Here, we discovered a novel bacterium, *Rhodoferax* sp. strain MIZ03, capable of both Fe(II) oxidation and Fe(III) reduction at circumneutral pH, and we report its physiological characteristics and complete genome sequence. The unexpected capability of this bacterium provides novel insights into the Fe cycle in the environment. Moreover, this bacterium will help to better understand the molecular mechanisms of microbial Fe redox cycling as a model organism.

The biogeochemical Fe cycle on the Earth’s surface is driven by phylogenetically and ecologically diverse Fe(II)-oxidizing microorganisms (FeOM) and Fe(III)-reducing microorganisms (FeRM) (1–4). Ferrous ion (Fe^2+^) is rapidly and abiotically oxidized and precipitated as Fe(III) oxide minerals at circumneutral pH under atmospheric conditions. However, the abiotic Fe(II) oxidation rate becomes slower under microaerobic conditions, and thus more Fe^2+^ is available for FeOM as the electron donor (3). Indeed, all of the known neutrophilic, O_2_-respirating FeOM, such as *Gallionella* spp., *Sideroxydans* spp., and *Mariprofundus* spp., are microaerophilic (3). The by-products from FeOM are solid Fe(III) minerals that can be reduced and redissolved to Fe^2+^ by FeRM, such as *Geobacter* spp. and *Shewanella* spp., as the electron acceptor under anaerobic conditions (4). The phylogenetic diversity of neutrophilic, microaerophilic FeOM is limitedly unveiled probably due to the technical difficulty of their cultivation. Accordingly, the molecular mechanisms of Fe(II) oxidation by neutrophilic FeOM are largely unclear (5–7). Nevertheless, a few candidate genes (e.g., *cyc2* and *mtoA*) involved in Fe(II) oxidation have been proposed (8, 9) and found in diverse microbial genomes/metagenome-assembled genomes (MAGs) (7), implying that neutrophilic FeOM are more phylogenetically diverse.

The genus *Rhodoferax* contains metabolically versatile species capable of photosynthesis, fermentation, and aerobic and/or anaerobic respiration. So far, Rhodoferax ferrireducens has been reported as the sole *Rhodoferax* species that can anaerobically grow by reduction of Fe(III) as the electron acceptor coupled with oxidation of organic acids and sugars as the electron donors (10, 11). A recent report of enrichment cultivation and metagenomics suggested the presence of H_2_-oxidizing and Fe(III)-reducing chemolithoautotrophic *Rhodoferax* spp. in a subglacial environment (12). The capability of Fe(II) oxidation by *Rhodoferax* spp. in iron mats has been proposed by environmental analyses of geochemistry and microbial communities (13). However, no pure culture of chemolithoautotrophic *Rhodoferax* spp. has been reported. Such chemolithoautotrophic *Rhodoferax* spp. potentially play a significant role in biogeochemical cycling of carbon and iron in the dark, organic-poor, subsurface environments where the inorganic energy sources are available. Here, we report a novel *Rhodoferax* isolate, designated strain MIZ03 (deposited in the Japan Collection of Microorganisms under the number JCM 34246), which grows chemolithoautotrophically at circumneutral pH using Fe(II), H_2_, or thiosulfate as the sole electron donor under (micro)aerobic conditions.

We collected an iron-rich floc sample in a freshwater wetland (see Fig. S1 in the supplemental material; 36°04′41″N, 140°05′24″E; 18°C, pH 6.5, <0.01% salinity) next Mizube Park, Tsukuba, Japan, in November 2018. Using the collected sample as the inoculum, we performed isolation of microaerophilic Fe(II)-oxidizing bacteria (FeOB) with a 96-well-plate gradient cultivation method as described previously (14). Details of experimental procedures are described in the supplemental material. We were successful in subcultivation of six positive cultures representing a typical iron-oxide band for microaerophilic FeOB cultures (15, 16). 16S rRNA gene clone analysis indicated the presence of known FeOB relatives (such as *Sideroxydans* and *Thiomonas* spp.) and a *Rhodoferax* relative in these cultures (Table S1). In this study, we focused on the *Rhodoferax*-containing culture. We subcultivated the *Rhodoferax*-containing culture on R2A plates and performed single-colony isolation repeatedly. Eventually, the *Rhodoferax* isolate, strain MIZ03, was obtained. The purity was checked using light microscopy, 16S rRNA gene analysis, and shotgun genome analysis. We confirmed that MIZ03, as well as the other FeOB strains obtained in this study, produced the iron-oxide band in the gradient culture tubes with an FeCO_3_ plug (Fig. S2), which resembles known microaerophilic freshwater FeOB (15, 16).

We determined the physiology and complete genome sequence of MIZ03 (Table S2). The phylogenomic tree (Fig. 1A) and the 16S rRNA gene similarity and average nucleotide identity values compared to the close relatives (Table S2) suggest that MIZ03 represents a novel species in the genus *Rhodoferax*. We tentatively propose the name Rhodoferax lithotrophicus for this strain. In the phylogenomic tree, MIZ03 was clustered with metagenome-assembled genomes (MAGs) recovered from deep subsurface groundwater environments (17, 18) but was distantly related to the two MAGs (KJH.1 and KJN.1) of the chemolithoautotrophic enrichment cultures from a subglacial environment (12) and to the genomes of any cultivates including *R. ferrireducens*.

**FIG 1:**
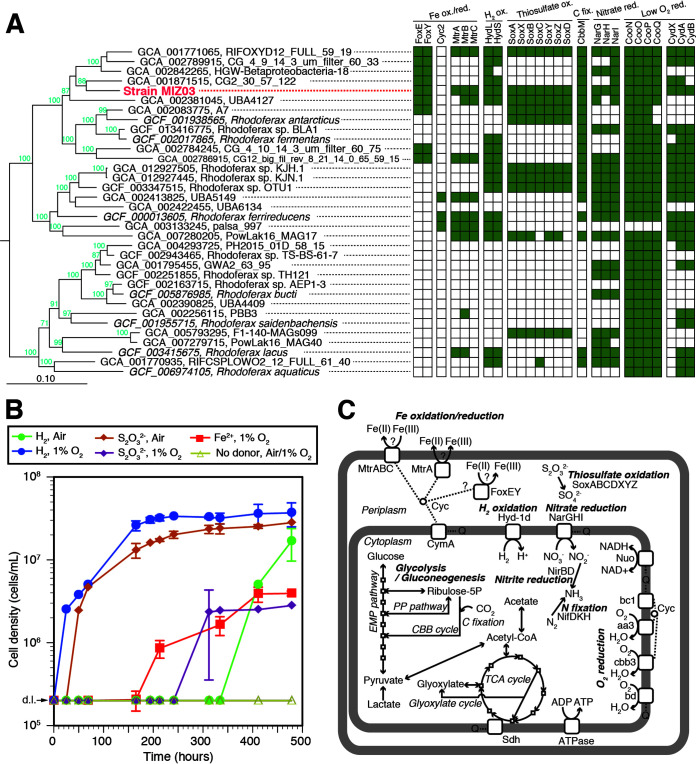
Genomics and chemolithoautotrophic growth of *Rhodoferax lithotrophicus* MIZ03. (A) Phylogenomic tree and gene content for microaerobic chemolithoautotrophy. A maximum-likelihood tree based on the alignment of 120 bacterial marker proteins is shown. Bootstrap values (only >50%) are indicated at the nodes. Curvibacter delicatus (GenBank accession no. GCF_001592265) was used as the outgroup (not shown). On the right side of panel A, the presence or absence (filled/opened squares) of genes for FoxEY, Cyc2, and MtrABC (Fe oxidation/reduction), large and small subunits of group I hydrogenase (H_2_ oxidation), SoxABCDXYZ (thiosulfate oxidation), CbbM (carbon fixation), NarGHI (nitrate reduction), CooNOPQ, and CydABX (aerobic respiration under low O_2_ concentration) is indicated for each genome/MAG. (B) Growth curves of MIZ03 growing under the 1% O_2_ and air conditions. Cell densities in the cultures with H_2_ and air (green-filled circles) or 1% O_2_ (blue-filled circles), with thiosulfate and air (orange-filled diamonds) or 1% O_2_ (purple-filled diamonds), with Fe(II) and 1% O_2_ (red-filled squares), and without any electron donors with air/1% O_2_ (yellow-opened diamonds), are shown. Error bars indicate the standard deviation of the mean of biological triplicates. For the thiosulfate-oxidizing cultures, an increase of sulfate concentration (from an initial concentration of 0.7 mM to 9.2 ± 0.3 mM at 21 days after inoculation) by thiosulfate oxidation was confirmed. For the H_2_-oxidizing cultures, consumption of H_2_ was observed (Fig. S3). For the experiments with cells, the initial cell densities were approximately 1 × 10^4^ cells/ml, as calculated from the cell numbers in the inoculum. All the data points under the detection limit (d.l.; 2 × 10^5^ cells/ml) are shown on the detection limit line. (C) Metabolic pathway of MIZ03 deduced from the annotated genome. A list of CDSs is shown in Table S4.

The optimum and range of growth temperature, pH, and salinity and the energy metabolism of MIZ03 and close relatives are summarized in Table S2 (more details for MIZ03 metabolism are shown in Table S3). For all the growth experiments for substrate utilization, the strain was transferred at least three times using the specific growth mode tested. MIZ03 grew chemoorganotrophically in R2A medium (containing various organic substances such as yeast extract, peptone, and glucose) under dark and (micro)aerobic conditions (1% O_2_ or air in the headspace). It also grew anaerobically by fermentation in R2A medium, as reported for *R. fermentans* (19).

Our cultivation experiments demonstrated that MIZ03 grew chemolithoautotrophically at circumneutral pH by oxidation of Fe(II), H_2_, or thiosulfate as the sole electron donor under (micro)aerobic conditions (Fig. 1B; Fig. S3 and S4). In addition, MIZ03 reduced nitrate and dissolved Fe(III) [i.e., Fe(III)-NTA (nitrilotriacetic acid) and Fe(III)-citrate] or a solid Fe(III) mineral (ferrihydrite), coupled with oxidation of organic substances in R2A medium as the electron donor under anaerobic conditions but did not reduce the other solid Fe(III) minerals tested (i.e., goethite, magnetite, and hematite) (Fig. S5; Tables S2 and S3). The previous studies reported that *R. ferrireducens* can reduce dissolved Fe(III) but not any solid Fe(III) minerals (10, 11). Although the reduction of ferrihydrite by MIZ03 enhanced its growth rate and yield (Fig. S5), it is unclear whether the growth enhancement is caused by an energetic or nutritional effects (i.e., increase of iron availability).

The genome content was consistent with the observed metabolism (Fig. 1C; Table S4). The MIZ03 genome encoded 87 protein-coding regions (CDSs) for *c*-type cytochrome with one or more heme-binding motifs (Table S5), including homologs of MtrA/MtoA, MtrC/MtoC, and FoxE, which have been reported as functional proteins for Fe redox reaction in known Fe(II) oxidizers (e.g., *Rhodobacter* and *Sideroxydans* spp.) and Fe(III) reducers (e.g., *Geobacter* and *Shewanella* spp.) as reviewed in reference 7. At present, it is unclear which (or even whether) homologs are involved in iron oxidation/reduction by MIZ03. No CDSs for Cyc2, which is thought to be involved in iron oxidation by microaerophilic FeOB (6, 9), were found. For H_2_ and thiosulfate oxidation and nitrate reduction, the MIZ03 genome encoded CDSs for respiratory H_2_-uptake [NiFe] hydrogenase (group 1d), SOX system, and nitrate reductase (NarGHI), respectively. In addition, it encoded a complete set of CDSs for the Calvin-Benson-Bassham cycle, including ribulose bisphosphate carboxylase/oxygenase (RubisCO) form II (CbbM) for autotrophy, and CDSs for *aa*_3_-, *cbb*_3_-, and *bd*-type terminal oxidases for (micro)aerobic respiration. No CDSs for photosynthesis were found.

Aerobic Fe(II) oxidation and anaerobic Fe(III) reduction by a single microorganism had been reported only in acidophiles (20). Some Fe(III) reducers can grow by anaerobic Fe(II) oxidation coupled with nitrate reduction (2), but not with aerobic respiration. For the first time, we demonstrate that, among all three domains of life, there is a single microorganism capable of microaerobic Fe(II) oxidation and anaerobic Fe(III) reduction at circumneutral pH. Such bifunctional microorganisms may have an ecological advantage in suboxic/anoxic environments by recycling of iron as the electron donor and acceptor. In addition to Fe(II), MIZ03 can grow chemolithoautotrophically using H_2_ or thiosulfate as the sole electron donor, which is consistent with its genome content. Notably, other *Rhodoferax* genomes/MAGs also encode the genes likely involved in the Fe(II)-, H_2_-, and thiosulfate-oxidizing chemolithoautotrophic growth under (micro)aerobic conditions (Fig. 1A). Indeed, the presence of such chemolithoautotrophic *Rhodoferax* spp. has been suggested in several environments (12, 13). Further cultivation and physiological tests may lead to discovering more diverse chemolithoautotrophic *Rhodoferax* spp. and to emphasizing their ecological significance. Finally, the unique *Rhodoferax* bacterium MIZ03 with the unexpected versatile metabolism, including Fe(II) oxidation and Fe(III) reduction, will open a new window on the ecophysiology and molecular mechanism of the Fe cycle as a model organism.

## Data availability.

The raw sequence data and the complete genome sequence of MIZ03 have been deposited in the DDBJ under the accession numbers DRA011244 and AP024238, respectively.
