# Supplementary material for: A Single Bacterium Capable of Oxidation and Reduction of Iron at Circumneutral pH
Source: Microbiol Spectr. 2021 Aug 25;9(1):10.1128/spectrum.00161-21. doi: 10.1128/spectrum.00161-21 (PMC8552755; doi:10.1128/spectrum.00161-21)
Supplement: SUPPLEMENTAL FILE 1 — Supplemental material. Download SPECTRUM00161-21_Supp_1_seq4.pdf, PDF file, 2.5 MB [file spectrum00161-21_supp_1_seq4.pdf]

Supplementary information

Materials and Methods

Sampling and isolation

Field sampling of iron-rich floc and measurement of pH and salinity were performed as previously reported (1). The collected sample was diluted in a modified Wolfe's mineral medium (MWMM) at pH 6.5, and used as inoculum for the 96-well plate cultivation method with agarose-stabilized MWMM as the top layer (180 µL per well) as previously described (1). In the present study, $FeCO_3$ instead of FeS was used as the bottom layer (20 µL per well) to avoid the growth of sulfur-oxidizing microorganisms. The 96-well culture plates were incubated at 25 ºC in the dark for over 2 weeks in gas-tight polycarbonate boxes with a gas mixture of N_2-CO_2-O_2 (79:20:1). Cultures with a typical iron-oxide band, which are generally observed in gradient culture tubes with microaerophilic iron-oxidizing bacteria (FeOB), were transferred to fresh gradient culture tubes (4 mL top layer with 500 µL bottom layer) and subcultivated. For the subcultures, 16S rRNA gene clone analysis was performed as reported previously (1). In brief, DNA was extracted from the subcultures using a FastDNA spin kit for soil (MP Biomedicals, Santa Ana, CA, USA), and 16S rRNA genes were amplified with the primers Bac27F and Uni1492R. The PCR products were cloned and sequenced.

Colony picking from R2A plate cultures were performed to obtain *Rhodoferax*- and *Thiomonas*-related isolates. Aliquots of the above subcultures were streaked on agar-solidified R2A plates, and the plates were incubated at 25 ºC under the air atmosphere. Distinct colonies were picked and re-streaked on R2A plates, and the plates were incubated as described above. This isolation process was repeated at least three times. To isolate *Sideroxydans* relatives, we performed limiting dilution and cultivation using the gradient culture tubes at least five times.

Physiological characterization

The strain MIZ03 isolated on R2A cultivation was used for the following physiological characterization. Range and optimum of growth temperature, pH and NaCl concentration for the isolated strain MIZ03 were determined using cultures in liquid R2A medium under the air atmosphere. The growth was checked by measurement of optical density (OD) at 660 nm using a miniphoto 518R (Taitec, Tokyo, Japan). To determine the growth temperature, its cultivation at pH 7

was tested at 4, 10, 15, 20, 25, 30, 35, 40, and 45 ºC. To determine the growth pH, its cultivation at 20 ºC was tested at pH 4.0, 5.0, 5.5, 6.0, 6.5, 7.0, 7.5, 8.0, 9.0, 10.0 and 11.0. To determine the growth NaCl concentration, its cultivation at 20 ºC and pH 6.5 was tested at 0, 0.25, 0.5, 0.75, 1.0, 2.0, and 3.0%, respectively.

Using the liquid MWMM as the base medium, the growth of MIZ03 was tested on inorganic substrates under the optimum condition (20 ºC and pH 6.5). For chemolithoautotrophic iron- or sulfur-oxidizing growth, $FeSO_4$ (final concentration, 10 mM), $Na_2S_2O_3$ (10 mM), Na_2SO_3 (10 mM), or S^0 (0.5 g/L) was added as the electron donor to the base medium, respectively. For hydrogen-oxidizing growth, H_2 gas (5% in the headspace) was added as the electron donor. For chemoorganotrophic growth, organic substrates in R2A (3.2 g/L) were added as the electron donor. As the electron acceptor, O_2 (1% with a gas mixture of N_2-CO_2 (79:20) or air in the headspace), Fe(III)-NTA (nitrilotriacetic acid) (10 mM), Fe(III)-citrate (10 mM), ferrihydrite (10 mM), goethite (1.0 g/L), hematite (1.0 g/L), magnetite (1.0 g/L), or $NaNO_3$ (10 mM) was tested. The ferrihydrite (amorphous Fe(III) oxyhydroxide) was prepared as previously described (2). The other solid Fe(III) minerals were purchased from Nacalai Tesque, Inc. (Kyoto, Japan). For fermentative growth, none of the electron acceptors were added. For all the growth experiments described above, the strain was transferred at least three times using the specific growth mode tested. Cell density in cultures was determined by measurement of optical density at 660 nm (OD_{660}) or by direct cell counting using a BX53 phase-contrast microscopy (Olympus, Tokyo, Japan).

The Fe^{2+} concentration in liquid media was measured by ferrozine assay (1). The NO_2^- and SO_4^{2-} concentrations were measured using a DR1900 spectrophotometer (Hach, Colorado, USA) by the NitriVer 2 and SulfaVer 4 methods. The H_2 in the headspace were analyzed using a gas chromatograph (GC-2014AT; Shimadzu, Kyoto, Japan) with a Shincarbon-ST 50/80 column (Shinwa Chemical Industries, LTD., Kyoto, Japan) and a thermal conductivity detector. The carrier gas was N_2 at a flow rate of 30 ml/min and the column temperature was 90 °C.

Genome analysis

The complete genome sequence of MIZ03 was determined as previously reported (3). In brief, we extracted DNA from MIZ03 cells in 100 mL of liquid R2A medium, and sequenced on a MiSeq platform (300 bp paired-end; Illumina, USA) and on a MinION device (Oxford Nanopore

Technologies [ONT], United Kingdom) with an R9.4 flow cell (ONT). Sequencing libraries were constructed using a QIAseq FX DNA library kit (Qiagen, Germany) for the Illumina sequencing and a rapid sequencing kit (SQK-RAD004, ONT) for the ONT sequencing. Quality filtering of the obtained reads using MetaWRAP (4) and NanoFilt (5), hybrid assembling of the high-quality long and short reads using Unicycler (6), and gene prediction and annotation using Prokka (7), RAST (8), and DFAST (9), were performed as previously reported (10). The annotation was manually curated based on the results from KEGG (11), InterProScan (12), eggNOG (13), HydDB (14), and FeGenie (15). Subcellular location of proteins was predicted by PSORTb (16). A phylogenomic tree of concatenated 120 marker protein sequences for the domain *Bacteria*, which have been defined in Genome Taxonomy Database Toolkit (GTDB-Tk) (17), was constructed as previously reported (18). In brief, the alignment provided by GTDB-Tk was trimmed using trimAl (19) with the '-automated1' option, and then used for the maximum-likelihood tree reconstruction by RaxML (20) with the GTRGAMMA model.

To find Cyc2 homologs, BLASTp search (homology-based), in addition to FeGenie (hidden Markov models (HMMs)-based) analysis as described above, was performed against the MIZ03 genome and the NCBI Reference protein (refseq_protein) database using Cyc2 homologs of known iron-oxidizing bacteria, i.e., *Mariprofundus ferrooxydans* (AKN35166), *Sideroxydans lithotrophicus* (ADE10507), and *Acidithiobacillus ferrooxidans* (ACH84943), as queries.

References

1. Kato S, Chan C, Itoh T, Ohkuma M. 2013. Functional gene analysis of freshwater iron-rich flocs at circumneutral pH and isolation of a stalk-forming microaerophilic iron-oxidizing bacterium. Appl Environ Microbiol 79:5283-90.
2. Lovley DR, Phillips EJ. 1986. Organic matter mineralization with reduction of ferric iron in anaerobic sediments. Appl Environ Microbiol 51:683-9.
3. Kato S, Yuki M, Itoh T, Ohkuma M. 2018. Complete genome sequence of *Ferriphaselus amnicola* strain OYT1, a neutrophilic, stalk-forming, iron-oxidizing bacterium. Microbiol Resour Announc 7:e00911-18.
4. Uritskiy GV, DiRuggiero J, Taylor J. 2018. MetaWRAP-a flexible pipeline for genome-resolved metagenomic data analysis. Microbiome 6:158.

5. De Coster W, D'Hert S, Schultz DT, Cruts M, Van Broeckhoven C. 2018. Nanopack: Visualizing and processing long-read sequencing data. Bioinformatics 34:2666-2669.

6. Wick RR, Judd LM, Gorrie CL, Holt KE. 2017. Unicycler: Resolving bacterial genome assemblies from short and long sequencing reads. PLoS Comput Biol 13:e1005595.

7. Seemann T. 2014. Prokka: Rapid prokaryotic genome annotation. Bioinformatics 30:2068-9.

8. Aziz R, Bartels D, Best A, DeJongh M, Disz T, Edwards R, Formsma K, Gerdes S, Glass E, Kubal M, Meyer F, Olsen G, Olson R, Osterman A, Overbeek R, McNeil L, Paarmann D, Paczian T, Parrello B, Pusch G, Reich C, Stevens R, Vassieva O, Vonstein V, Wilke A, Zagnitko O. 2008. The RAST server: Rapid annotations using subsystems technology. BMC Genomics 9:75.

9. Tanizawa Y, Fujisawa T, Nakamura Y. 2018. DFAST: A flexible prokaryotic genome annotation pipeline for faster genome publication. Bioinformatics 34:1037-1039.

10. Kato S, Itoh T, Ohkuma M. 2020. Complete genome sequence of *Athalassotoga saccharophila* strain NAS-01, a deep-branching thermophilic lineage in the phylum *Thermotogae*. Microbiol Resour Announc 9:e00322-20.

11. Ogata H, Goto S, Sato K, Fujibuchi W, Bono H, Kanehisa M. 1999. KEGG: Kyoto encyclopedia of genes and genomes. Nucleic Acids Res 27:29-34.

12. Zdobnov EM, Apweiler R. 2001. InterProScan--an integration platform for the signature-recognition methods in InterPro. Bioinformatics 17:847-8.

13. Huerta-Cepas J, Szklarczyk D, Heller D, Hernandez-Plaza A, Forslund SK, Cook H, Mende DR, Letunic I, Rattei T, Jensen LJ, von Mering C, Bork P. 2019. eggNOG 5.0: A hierarchical, functionally and phylogenetically annotated orthology resource based on 5090 organisms and 2502 viruses. Nucleic Acids Res 47:D309-D314.

14. Sondergaard D, Pedersen CN, Greening C. 2016. HydDB: A web tool for hydrogenase classification and analysis. Sci Rep 6:34212.

15. Garber AI, Nealson KH, Okamoto A, McAllister SM, Chan CS, Barco RA, Merino N. 2020. FeGenie: A comprehensive tool for the identification of iron genes and iron gene neighborhoods in genome and metagenome assemblies. Front Microbiol 11:37.

16. Yu NY, Wagner JR, Laird MR, Melli G, Rey S, Lo R, Dao P, Sahinalp SC, Ester M, Foster LJ, Brinkman FS. 2010. PSORTb 3.0: Improved protein subcellular localization prediction with

refined localization subcategories and predictive capabilities for all prokaryotes. Bioinformatics 26:1608-15.

17. Chaumeil PA, Mussig AJ, Hugenholtz P, Parks DH. 2020. GTDB-Tk: A toolkit to classify genomes with the genome taxonomy database. Bioinformatics 36:1925–1927.
18. Kato S, Ohnishi M, Nagamori M, Yuki M, Takashina T, Ohkuma M, Itoh T. 2021. *Conexivisphaera calida* gen. nov., sp. nov., a thermophilic sulfur- and iron-reducing archaeon, and proposal of *Conexivisphaeraceae* fam. nov., *Conexivisphaerales* ord. nov., and *Conexivisphaeria* class. nov. in the phylum *Thaumarchaeota*. Int J Syst Evol Microbiol 71:doi: 10.1099/ijsem.0.004595.
19. Capella-Gutierrez S, Silla-Martinez JM, Gabaldon T. 2009. trimAl: A tool for automated alignment trimming in large-scale phylogenetic analyses. Bioinformatics 25:1972-3.
20. Stamatakis A. 2014. RAxML version 8: A tool for phylogenetic analysis and post-analysis of large phylogenies. Bioinformatics 30:1312-3.
21. Finneran KT, Johnsen CV, Lovley DR. 2003. *Rhodoferax ferrireducens* sp. nov., a psychrotolerant, facultatively anaerobic bacterium that oxidizes acetate with the reduction of Fe(III). Int J Syst Evol Microbiol 53:669-673.
22. Chaudhuri SK, Lovley DR. 2003. Electricity generation by direct oxidation of glucose in mediatorless microbial fuel cells. Nat Biotechnol 21:1229-32.
23. Hiraishi A, Hoshino Y, Satoh T. 1991. *Rhodoferax fermentans* gen. nov., sp. nov., a phototrophic purple nonsulfur bacterium previously referred to as the "*Rhodocyclus gelatinosus*-like" group. Archives of Microbiology 155:330-336.
24. Madigan MT, Jung DO, Woese CR, Achenbach LA. 2000. *Rhodoferax antarcticus* sp. nov., a moderately psychrophilic purple nonsulfur bacterium isolated from an Antarctic microbial mat. Arch Microbiol 173:269-77.
25. Zhou, Tan X, Zhang W, Chen HY, Fan QM, He XL, Lv J. 2019. *Rhodoferax bucti* sp. nov., isolated from fresh water. Int J Syst Evol Microbiol 69:3903-3909.
26. Park M, Song J, Nam GG, Cho JC. 2019. *Rhodoferax lacus* sp. nov., isolated from a large freshwater lake. Int J Syst Evol Microbiol 69:3135-3140.
27. Li T, Zhuo Y, Jin CZ, Wu X, Ko SR, Jin FJ, Ahn CY, Oh HM, Lee HG, Jin L. 2020. Genomic insights into a novel species *Rhodoferax aquaticus* sp. nov., isolated from freshwater. Int J Syst

Evol Microbiol 70:4653-4660.

28. Kaden R, Sproer C, Beyer D, Krolla-Sidenstein P. 2014. *Rhodoferax saidenbachensis* sp. nov., a psychrotolerant, very slowly growing bacterium within the family *Comamonadaceae*, proposal of appropriate taxonomic position of *Albidiferax ferrireducens* strain T118^T in the genus *Rhodoferax* and emended description of the genus *Rhodoferax*. Int J Syst Evol Microbiol 64:1186-1193.

SI Figure legends

Fig. S1. Iron-rich flocs at the sampling site. (A) On-site photo of sampling site and (B) enlarged view. Orangish iron-oxide flocs developed in the wetland. In the floc areas, blackish spots (indicated by yellow arrows) were patchily observed. (C) Phase-contrast and (D) fluorescence microscopic images of the floc sample in the same view area. *Leptothrix*-like sheath structures were observed. Cells were stained with SYBR Green I. Scale bars, 50 µm.

Fig. S2. Gradient culture tubes. (A) Illustration and (B) photos of gradient culture tubes at 7 days after inoculation. A sharp iron-oxide band was observed near the surface in the pure cultures of MIZ01 and MIZ03, and the co-culture of MIZ02 with MIZ09. In contrast, the iron-oxide band was broader in the pure culture of MIZ09.

Fig. S3. Growth of MIZ03 using H_2 as the sole electron donor. (A) Measurement of H_2 and (B) growth curve in the microaerobic (1% O_2) incubation experiments with cells and H_2 (triangle), with cells but without H_2 (circle), and without cells but with H_2 (square). Decrease of H_2 concentration was observed only in the experiments with cells and H_2, indicating biotic H_2 oxidation by MIZ03. Error bars indicate the standard deviation of the mean of biological triplicate. Black allows indicate the additional inputs of $H_{2.}$ For the experiments with cells, the initial cell densities were approximately 1×10^4 cells/mL, as calculated from the cell numbers in the inoculum. All the data points under the detection limit (d.l.; 2×10^5 cells/mL) is shown on the detection limit line.

Fig. S4. Microscopic images of MIZ03 cells. (A) Phase-contrast and (B) fluorescence microscopic images of cells in the microaerobic (1% O_2) cultures with Fe(II) at 14 days after inoculation. In many cases, cells were attached to iron oxides. (C) Phase-contrast and (D) fluorescence microscopic images of cells in the microaerobic (1% O_2) cultures with H_2 at 10 days after inoculation. (E) Phase-contrast and (F) fluorescence microscopic images of cells in the aerobic (air) cultures with thiosulfate at 10 days after inoculation. For each culture, phase-contrast and fluorescence microscopic images were taken in the same view area. Cells were stained with SYBR Green I. Scale bars, 20 µm.

Fig. S5. Anaerobic growth of MIZ03 by ferrihydrite reduction. Growth curves of MIZ03 growing

in R2A media with ferrihydrite (red circle) and without ferrihydrite (green circle) under the anaerobic conditions are shown. Concentration of Fe^{2+} in the cultures containing ferrihydrite with cells (blue square) and without cells (orange square) are also shown. pH was stable at 6.4-6.7 during the cultivation. Error bars indicate the standard deviation of the mean of biological triplicate.

SI Figures

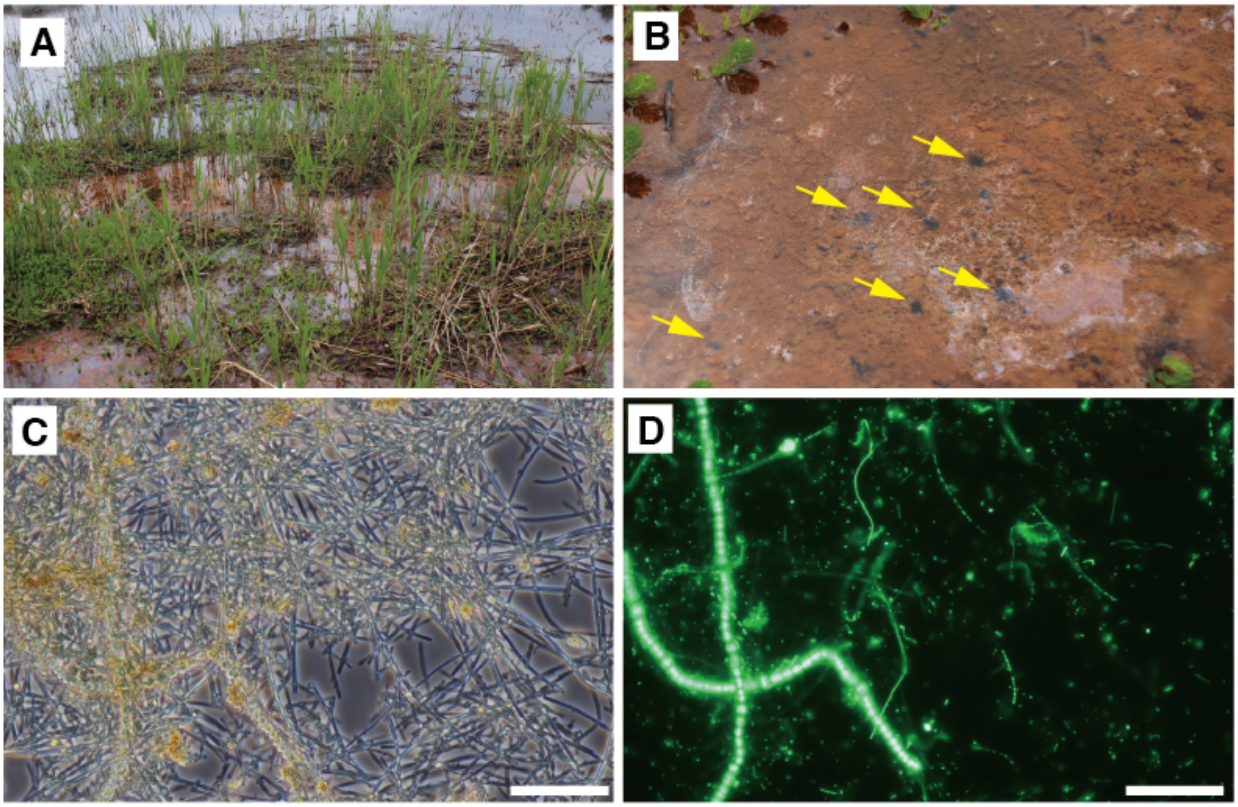

Fig. S1. Iron-rich flocs at the sampling site. (A) On-site photo of sampling site and (B) enlarged view. Orangish iron-oxide flocs developed in the wetland. In the floc areas, blackish spots (indicated by yellow arrows) were patchily observed. (C) Phase-contrast and (D) fluorescence microscopic images of the floc sample in the same view area. *Leptothrix*-like sheath structures were observed. Cells were stained with SYBR Green I. Scale bars, 50 µm.

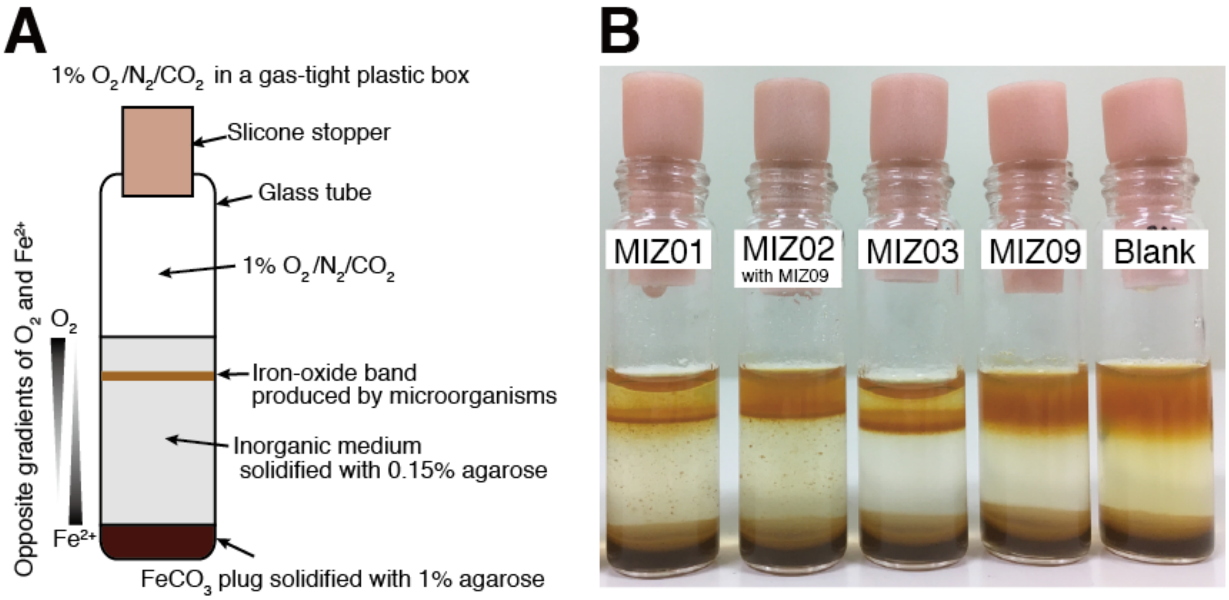

Fig. S2. Gradient culture tubes. (A) Illustration and (B) photos of gradient culture tubes at 7 days after inoculation. A sharp iron-oxide band was observed near the surface in the pure cultures of MIZ01 and MIZ03, and the co-culture of MIZ02 with MIZ09. In contrast, the iron-oxide band was broader in the pure culture of MIZ09.

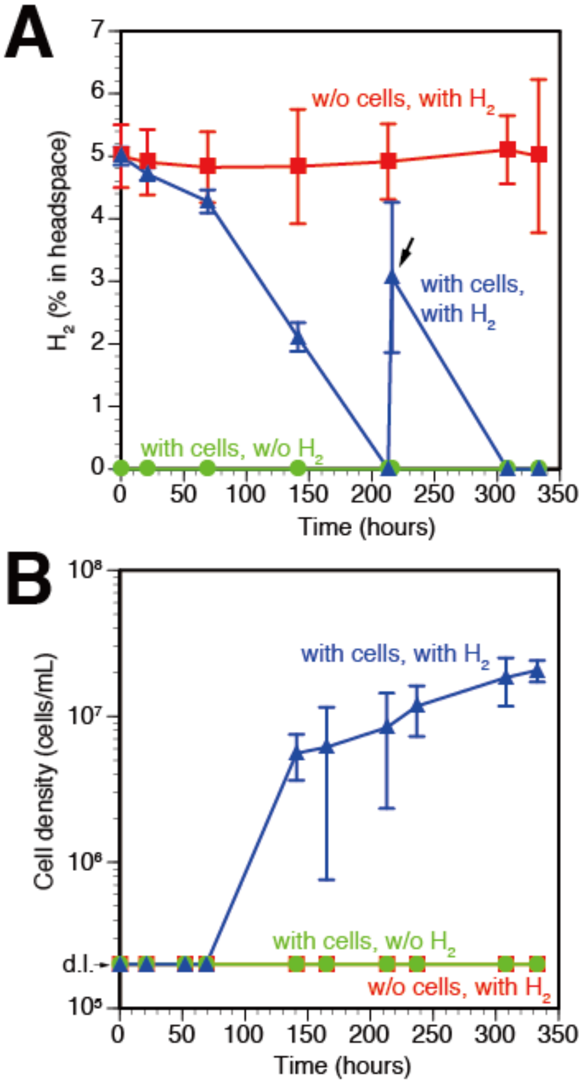

Fig. S3. Growth of MIZ03 using H_2 as the sole electron donor. (A) Measurement of H_2 and (B) growth curve in the microaerobic (1% O_2) incubation experiments with cells and H_2 (triangle), with cells but without H_2 (circle), and without cells but with H_2 (square). Decrease of H_2 concentration was observed only in the experiments with cells and H_2, indicating biotic H_2 oxidation by MIZ03. Error bars indicate the standard deviation of the mean of biological triplicate. Black allows indicate the additional inputs of H_2. For the experiments with cells, the initial cell densities were approximately 1×10^4 cells/mL, as calculated from the cell numbers in the inoculum. All the data points under the detection limit (d.l.; 2×10^5 cells/mL) is shown on the detection limit line.

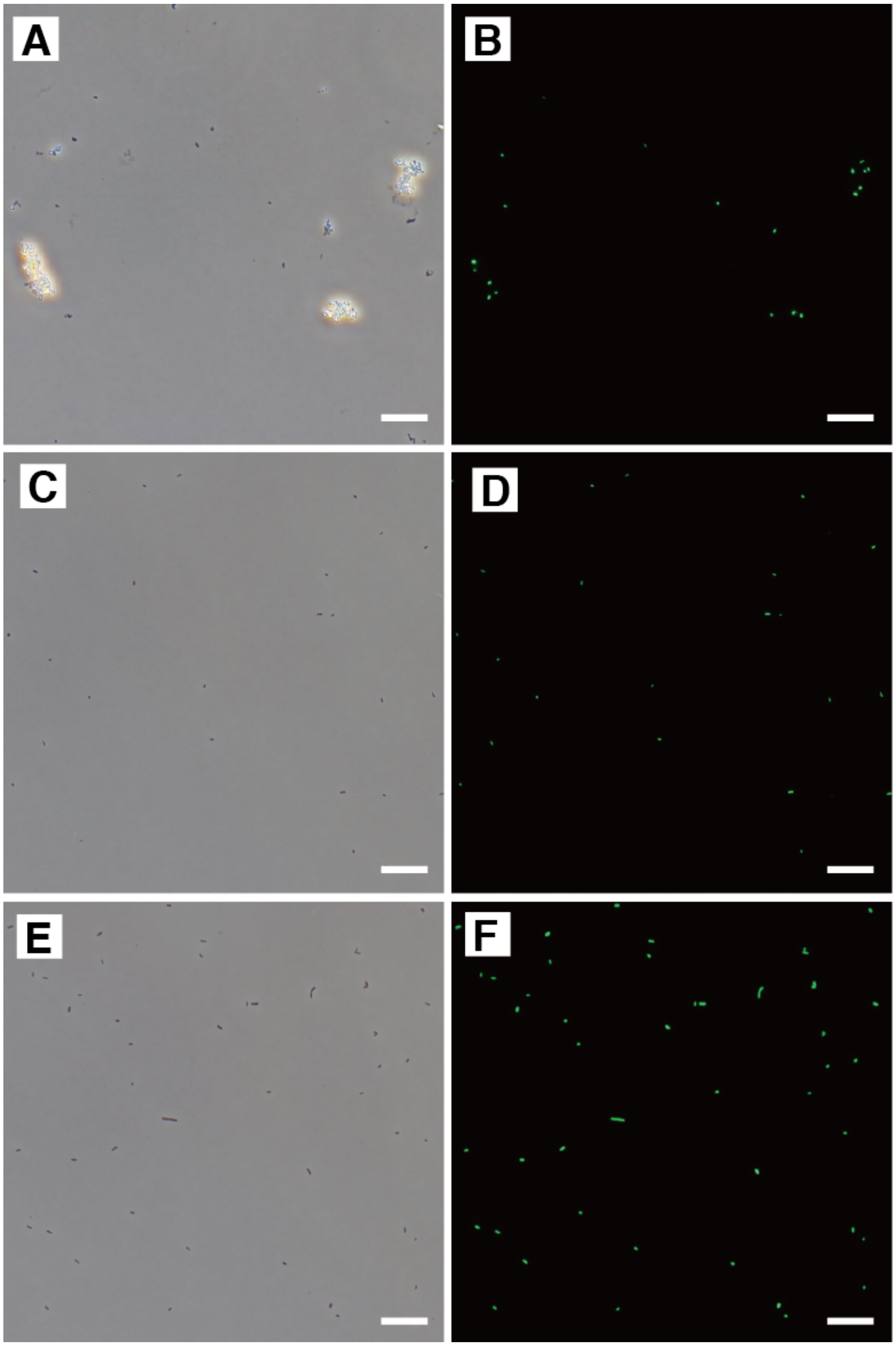

Fig. S4. Microscopic images of MIZ03 cells. (A) Phase-contrast and (B) fluorescence microscopic images of cells in the microaerobic (1% O_2) cultures with Fe(II) at 14 days after inoculation. In many cases, cells were attached to iron oxides. (C) Phase-contrast and (D) fluorescence microscopic images of cells in the microaerobic (1% O_2) cultures with H_2 at 10 days after inoculation. (E) Phase-contrast and (F) fluorescence microscopic images of cells in the aerobic (air) cultures with thiosulfate at 10 days after inoculation. For each culture, phase-contrast and fluorescence microscopic images were taken in the same view area. Cells were stained with SYBR Green I. Scale bars, 20 µm.

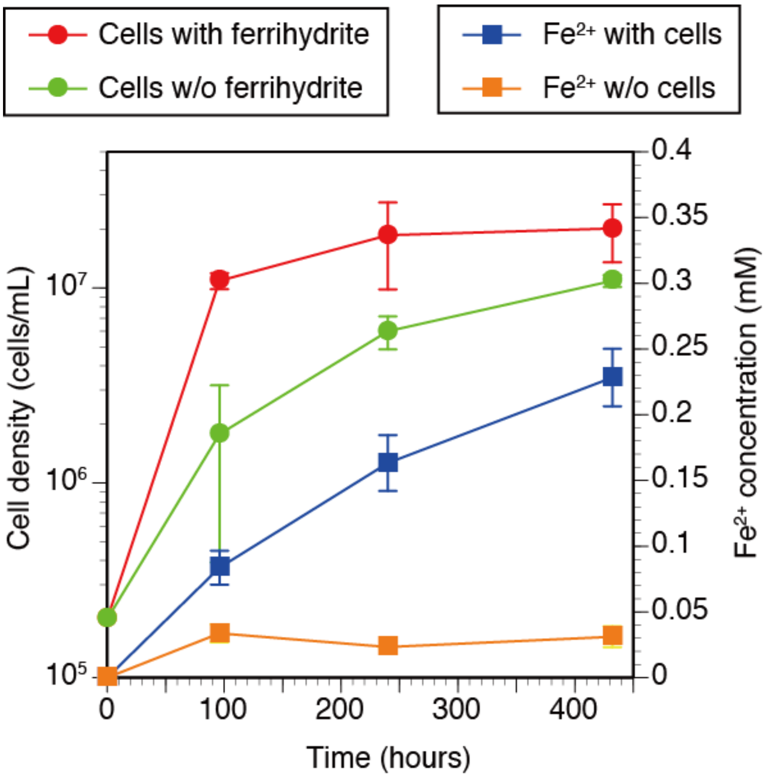

Fig. S5. Anaerobic growth of MIZ03 by ferrihydrite reduction. Growth curves of MIZ03 growing in R2A media with ferrihydrite (red circle) and without ferrihydrite (green circle) under the anaerobic conditions are shown. Concentration of Fe^{2+} in the cultures containing ferrihydrite with cells (blue square) and without cells (orange square) are also shown. pH was stable at 6.4-6.7 during the cultivation. Error bars indicate the standard deviation of the mean of biological triplicate.

Table S1. Microorganisms in gradient subcultures detected by 16S rRNA gene analysis

Culture ID	Detected microorganisms	Closest cultivated species	Accession no.	16S rRNA gene similarity (%)*
2-4 M	*Sideroxydans* -related strain MIZ01	*'Sideroxydans lithotrophicus'* str. ES-1	CP001965	98.25
2-1D	*Thiomonas* -related strain MIZ07	*'Thiomonas ferrovorans'* str. FB-6	JN885793	99.86
2-10B	***Rhodoferax*-related strain MIZ03**	*Rhodoferax ferrireducens* str. T118	CP000267	97.55
	Curvibacter-related strain MIZ04	*Curvibacter gracilis* str. DST51	MT254903	100.00
	Pinisolibacter-related strain MIZ10	*Pinisolibacter ravus* str. E9	KY087994	98.69
2-11D	*Thiomonas* -related strain MIZ08	*'Thiomonas ferrovorans'* str. FB-6	JN885793	99.86
	Asticcacaulis -related strain MIZ11	*Asticcacaulis solisilvae* str. CGM1-3EN	NR_109665	97.62
4-6C	*Thiomonas* -related strain MIZ06	*'Thiomonas ferrovorans'* str. FB-6	JN885793	99.86
4-11C	*Sideroxydans* -related strain MIZ02	*'Sideroxydans lithotrophicus'* str. ES-1	CP001965	98.96
	Thiomonas -related strain MIZ09	*'Thiomonas ferrovorans'* str. FB-6	JN885793	99.86

*Results from BLASTn against NCBI nr database using approx. 500 bp sequenced with Bac27F

Table S2. Summary of physiology and genomic feature of MIZ03 and *Rhodoferax* relatives

	R. lithotrophicus str. MIZ03	*Rhodoferax* species						
		R. ferrireducens	*R. fermentans*	*R. antarcticus*	*R. bucti*	*R. lacus*	*R. aquaticus*	*R. saidenbachensis*
Reference	This study	21,22	23	24	25	26	27	28
Growth conditions[a]								
Temperature (℃)	4–30 (20)	4–30 (25)	<37 (25–30)	0–25 (12–18)	15–35 (25)	4–30 (25)	4–30 (25–30)	4–30 (20)
pH	5.5–8.0 (6.5–7.0)	6.7–7.1 (7.0)	5.0–9.0 (6.5–7.0)	N.D.	5.0–10.0 (7.0)	5.0–8.0 (8.0)	7.0–9.0 (7.0)	6.0–9.0 (6.0)
Salinity (%NaCl)	0–0.75 (0)	N.D.[b]	<1.0	0–2.0 (0–0.1)	0–1.0 (0.5)	0–1.0 (0)	N.D.	0–0.9
Energy metabolism								
Aerobic lithotrophy	H_2, Fe(II), thiosulfate	N.D.	N.D.	N.D.	N.D.	N.D.	N.D.	N.D.
Aerobic organotrophy	+	+	+	+	+	+	+	+
Anaerobic reduction	Fe(III)-NTA, Fe(III)-citrate, ferrihydrite, nitrate	Fe(III)–NTA, Mn(IV) oxide, fumarate, nitrate	Fumarate	N.D.	Nitrate	Nitrate	-	-
Fermentation	+	-	+	-	-	-	N.D.	-
Phototrophy	N.D.	-	+	+	N.D.	-	N.D.	-
Genomic feature								
Number of contigs	1	1	2	20	8	72	1	1
Genome size (bp)	4,936,681	4,969,784	4,467,741	4,007,233	3,673,501	4,900,405	4,737,690	4,264,855
G+C content (%)	56.4	59.9	56.9	59.0	61.2	62.3	57.4	60.9
Number of CDSs	4757	4339	4176	3786	3488	4420	4367	4030
Number of tRNAs	54	45	53	47	42	44	49	46
Number of rRNAs	9	6	12	9	4	7	15	6
Average nucleotide identity (%)[c]	100	79.66	79.42	78.81	77.60	77.74	77.78	77.77
16S rRNA gene similarity (%)[c]	100	98.44	97.39	98.44	97.27	98.30	97.98	98.37
Accession no.	AP024238	CP000267	MTJN00000000	MSYM00000000	VAHD00000000	QFZK00000000	CP036282	CP019239

[a] Optimum values are indicated in parentheses; [b] N.D., not determined; [c] Values against MIZ03

Table S3. Growth test on various substrates

Electron donors	Electron acceptor /Fermentation	Growth
Chemolithoautotrophy		
H_2	O_2 (air)	+
H_2	O_2 (1%)	+
H_2	Nitrate	-
H_2	Fe(III)-NTA, Fe(III)-citrate, ferrihydrite	-
Fe(II)	O_2 (air)	-
Fe(II)	O_2 (1%)	+
Thiosulfate	O_2 (air)	+
Thiosulfate	O_2 (1%)	+
Sulfite	O_2 (air)	-
Sulfite	O_2 (1%)	-
Elemental sulfur	O_2 (air)	-
Elemental sulfur	O_2 (1%)	-
Chemoorganotrophy		
R2A[a]	O_2 (air)	+
R2A	O_2 (1%)	+
R2A	Fe(III)-NTA, Fe(III)-citrate, ferrihydrite	+[b]
R2A	Goethite, hematite, magnetite	-
R2A	Nitrate	+[c]
R2A	Fermentation[d]	+

[a]R2A conteins peptone, casamino acids, pyruvate, glucose, yeast extract, dextrose, and starch

[b]Production of Fe(II) was confirmed

[c]Production of nitrite was confirmed

[d]Without any addition of electron acceptors

Table S4. List of CDSs used in the metabolic prediction (Fig. 1C)

KEGG pathway/module	Definition	Locus tag
**Central carbohydrate metabolism**		
**Glycolysis (Embden-Meyerhof pathway)/Gluconeogenesis**		
K00134	glyceraldehyde 3-phosphate dehydrogenase [EC:1.2.1.12]	MIZ03_0076, MIZ03_3510
K00845	glucokinase [EC:2.7.1.2]	MIZ03_0574
K00873	pyruvate kinase [EC:2.7.1.40]	MIZ03_3898
K00927	phosphoglycerate kinase [EC:2.7.2.3]	MIZ03_3509, MIZ03_4323
K01086	fructose-1,6-bisphosphatase I / sedoheptulose-1,7-bisphosphatase [EC:3.1.3.11 3.1.3.37]	MIZ03_3514
K01596	phosphoenolpyruvate carboxykinase (GTP) [EC:4.1.1.32]	MIZ03_0098
K01624	fructose-bisphosphate aldolase, class II [EC:4.1.2.13]	MIZ03_3507, MIZ03_3896
K01689	enolase [EC:4.2.1.11]	MIZ03_2473
K01803	triosephosphate isomerase (TIM) [EC:5.3.1.1]	MIZ03_1669
K01810	glucose-6-phosphate isomerase [EC:5.3.1.9]	MIZ03_1125
K01834	2,3-bisphosphoglycerate-dependent phosphoglycerate mutase [EC:5.4.2.11]	MIZ03_1927
K03841	fructose-1,6-bisphosphatase I [EC:3.1.3.11]	MIZ03_3349
K15634	2,3-bisphosphoglycerate-dependent phosphoglycerate mutase [EC:5.4.2.11]	MIZ03_0563
K21071	ATP-dependent phosphofructokinase / diphosphate-dependent phosphofructokinase [EC:2.7.1.11 2.7.1.90]	MIZ03_0048, MIZ03_0178
**Pyruvate oxidation**		
K00163	pyruvate dehydrogenase E1 component [EC:1.2.4.1]	MIZ03_0681, MIZ03_2449
K00169	pyruvate ferredoxin oxidoreductase alpha subunit [EC:1.2.7.1]	MIZ03_1997
K00170	pyruvate ferredoxin oxidoreductase beta subunit [EC:1.2.7.1]	MIZ03_1998
K00172	pyruvate ferredoxin oxidoreductase gamma subunit [EC:1.2.7.1]	MIZ03_1996
K00382	dihydrolipoamide dehydrogenase [EC:1.8.1.4]	MIZ03_2451, MIZ03_2547
K00627	pyruvate dehydrogenase E2 component (dihydrolipoamide acetyltransferase) [EC:2.3.1.12]	MIZ03_2450
**Citrate cycle (TCA cycle)**		
K00024	malate dehydrogenase [EC:1.1.1.37]	MIZ03_3475
K00030	isocitrate dehydrogenase (NAD+) [EC:1.1.1.41]	MIZ03_0756
K00031	isocitrate dehydrogenase [EC:1.1.1.42]	MIZ03_1229, MIZ03_1241
K00164	2-oxoglutarate dehydrogenase E1 component [EC:1.2.4.2]	MIZ03_2549
K00174	2-oxoglutarate/2-oxoacid ferredoxin oxidoreductase subunit alpha [EC:1.2.7.3 1.2.7.11]	MIZ03_0171
K00175	2-oxoglutarate/2-oxoacid ferredoxin oxidoreductase subunit beta [EC:1.2.7.3 1.2.7.11]	MIZ03_0172
K00239	succinate dehydrogenase / fumarate reductase, flavoprotein subunit [EC:1.3.5.1 1.3.5.4]	MIZ03_3479
K00240	succinate dehydrogenase / fumarate reductase, iron-sulfur subunit [EC:1.3.5.1 1.3.5.4]	MIZ03_3480
K00241	succinate dehydrogenase / fumarate reductase, cytochrome b subunit	MIZ03_3477
K00242	succinate dehydrogenase / fumarate reductase, membrane anchor subunit	MIZ03_3478
K00382	dihydrolipoamide dehydrogenase [EC:1.8.1.4]	MIZ03_2451, MIZ03_2547
K00658	2-oxoglutarate dehydrogenase E2 component (dihydrolipoamide succinyltransferase) [EC:2.3.1.61]	MIZ03_2548
K01647	citrate synthase [EC:2.3.3.1]	MIZ03_3482
K01676	fumarate hydratase, class I [EC:4.2.1.2]	MIZ03_2268
K01679	fumarate hydratase, class II [EC:4.2.1.2]	MIZ03_2941
K01682	aconitate hydratase 2 / 2-methylisocitrate dehydratase [EC:4.2.1.3 4.2.1.99]	MIZ03_3472
K01902	succinyl-CoA synthetase alpha subunit [EC:6.2.1.5]	MIZ03_4505
K01903	succinyl-CoA synthetase beta subunit [EC:6.2.1.5]	MIZ03_4504
K18118	succinyl-CoA:acetate CoA-transferase [EC:2.8.3.18]	MIZ03_3167
**Pentose phosphate pathway**		
K00615	transketolase [EC:2.2.1.1]	MIZ03_0075, MIZ03_3512
K00616	transaldolase [EC:2.2.1.2]	MIZ03_1124
K01783	ribulose-phosphate 3-epimerase [EC:5.1.3.1]	MIZ03_3515
K01807	ribose 5-phosphate isomerase A [EC:5.3.1.6]	MIZ03_2667
**PRPP biosynthesis**		
K00948	ribose-phosphate pyrophosphokinase [EC:2.7.6.1]	MIZ03_4146
**Glyoxylate cycle**		
K00024	malate dehydrogenase [EC:1.1.1.37]	MIZ03_3475
K01637	isocitrate lyase [EC:4.1.3.1]	MIZ03_2051
K01638	malate synthase [EC:2.3.3.9]	MIZ03_1170, MIZ03_1178
K01647	citrate synthase [EC:2.3.3.1]	MIZ03_3482
K01682	aconitate hydratase 2 / 2-methylisocitrate dehydratase [EC:4.2.1.34.2.1.99]	MIZ03_3472
**Phosphate acetyltransferase-acetate kinase pathway**		
K00625	phosphate acetyltransferase [EC:2.3.1.8]	MIZ03_3653
K00925	acetate kinase [EC:2.7.2.1]	MIZ03_3652
K13788	phosphate acetyltransferase [EC:2.3.1.8]	MIZ03_4659
**Lactate oxidation**		
K00101	L-lactate dehydrogenase (cytochrome) [EC:1.1.2.3]	MIZ03_0549
K00102	D-lactate dehydrogenase (cytochrome) [EC:1.1.2.4]	MIZ03_0228
**Carbon fixation**		
**Reductive pentose phosphate cycle (Calvin cycle)**		
K00134	glyceraldehyde 3-phosphate dehydrogenase [EC:1.2.1.12]	MIZ03_0076, MIZ03_3510
K00615	transketolase [EC:2.2.1.1]	MIZ03_0075, MIZ03_3512
K00855	phosphoribulokinase [EC:2.7.1.19]	MIZ03_3513
K00927	phosphoglycerate kinase [EC:2.7.2.3]	MIZ03_3509, MIZ03_4323
K01086	fructose-1,6-bisphosphatase I / sedoheptulose-1,7-bisphosphatase [EC:3.1.3.11 3.1.3.37]	MIZ03_3514
K01601	ribulose-bisphosphate carboxylase large chain [EC:4.1.1.39]	MIZ03_0927
K01624	fructose-bisphosphate aldolase, class II [EC:4.1.2.13]	MIZ03_3507, MIZ03_3896
K01807	ribose 5-phosphate isomerase A [EC:5.3.1.6]	MIZ03_2667
K03841	fructose-1,6-bisphosphatase I [EC:3.1.3.11]	MIZ03_3349
**Energy metabolism**		
**Iron oxidation/reduction**		
MtrC/MtoC	metal-reductase/oxidase subunit C	MIZ03_1615
MtrA/MtoA	metal-reductase/oxidase subunit A	MIZ03_1616, MIZ03_1878
MtrB/MtoB	metal-reductase/oxidase subunit B	MIZ03_1617
Cyc1	c-type cytochrome 1	MIZ03_1880, MIZ03_1881
CymA	tetraheme cytochrome c	MIZ03_0045
FoxY	Fe oxidase subunit Y	MIZ03_4239
FoxE	Fe oxidase subunit E	MIZ03_4240
**Hydrogen oxidation**		
K06281	hydrogenase large subunit [EC:1.12.99.6]	MIZ03_4644
K06282	hydrogenase small subunit [EC:1.12.99.6]	MIZ03_4645
**Thiosulfate oxidation**		
K17222	L-cysteine S-thiosulfotransferase [EC:2.8.5.2] (SoxA)	MIZ03_2070
K17223	L-cysteine S-thiosulfotransferase [EC:2.8.5.2] (SoxY)	MIZ03_2069
K17224	S-sulfosulfanyl-L-cysteine sulfohydrolase [EC:3.1.6.20] (SoxB)	MIZ03_2068
K17225	sulfane dehydrogenase subunit SoxC	MIZ03_2074
K17226	sulfur-oxidizing protein SoxY	MIZ03_2072
K17227	sulfur-oxidizing protein SoxZ	MIZ03_2071
K22622	S-disulfanyl-L-cysteine oxidoreductase SoxD [EC:1.8.2.6]	MIZ03_2073
**Nitrogen metabolism**		
K00362	nitrite reductase (NADH) large subunit [EC:1.7.1.15]	MIZ03_3188
K00363	nitrite reductase (NADH) small subunit [EC:1.7.1.15]	MIZ03_3186
K00370	nitrate reductase / nitrite oxidoreductase, alpha subunit [EC:1.7.5.1 1.7.99.-]	MIZ03_1761
K00371	nitrate reductase / nitrite oxidoreductase, beta subunit [EC:1.7.5.1 1.7.99.-]	MIZ03_1760
K00374	nitrate reductase gamma subunit [EC:1.7.5.1 1.7.99.-]	MIZ03_1758, MIZ03_2113
K02586	nitrogenase molybdenum-iron protein alpha chain [EC:1.18.6.1]	MIZ03_0739
K02588	nitrogenase iron protein NifH	MIZ03_0740
K02591	nitrogenase molybdenum-iron protein beta chain [EC:1.18.6.1]	MIZ03_0738
**ATP synthesis**		

**NADH:quinone oxidoreductase**		
K00330	NADH-quinone oxidoreductase subunit A [EC:7.1.1.2]	MIZ03_1672
K00331	NADH-quinone oxidoreductase subunit B [EC:7.1.1.2]	MIZ03_1673
K00332	NADH-quinone oxidoreductase subunit C [EC:7.1.1.2]	MIZ03_1674
K00333	NADH-quinone oxidoreductase subunit D [EC:7.1.1.2]	MIZ03_1675
K00334	NADH-quinone oxidoreductase subunit E [EC:7.1.1.2]	MIZ03_1676
K00335	NADH-quinone oxidoreductase subunit F [EC:7.1.1.2]	MIZ03_1677
K00336	NADH-quinone oxidoreductase subunit G [EC:7.1.1.2]	MIZ03_1678
K00337	NADH-quinone oxidoreductase subunit H [EC:7.1.1.2]	MIZ03_1679
K00338	NADH-quinone oxidoreductase subunit I [EC:7.1.1.2]	MIZ03_1680
K00339	NADH-quinone oxidoreductase subunit J [EC:7.1.1.2]	MIZ03_1681
K00340	ADH-quinone oxidoreductase subunit K [EC:7.1.1.2]	MIZ03_1682
K00341	NADH-quinone oxidoreductase subunit L [EC:7.1.1.2]	MIZ03_1683
K00342	NADH-quinone oxidoreductase subunit M [EC:7.1.1.2]	MIZ03_1684
K00343	NADH-quinone oxidoreductase subunit N [EC:7.1.1.2]	MIZ03_1685
**Cytochrome bc1 complex respiratory unit**		
K00411	ubiquinol-cytochrome c reductase iron-sulfur subunit [EC:7.1.1.8]	MIZ03_3532
K00412	ubiquinol-cytochrome c reductase cytochrome b subunit	MIZ03_3531
K00413	ubiquinol-cytochrome c reductase cytochrome c1 subunit	MIZ03_3530
**Cytochrome c oxidase**		
K02274	cytochrome c oxidase subunit I [EC:7.1.1.9]	MIZ03_3746
K02275	cytochrome c oxidase subunit II [EC:7.1.1.9]	MIZ03_3747
K02276	cytochrome c oxidase subunit III [EC:7.1.1.9]	MIZ03_3743
**Cytochrome bd ubiquinol oxidase**		
K00424	cytochrome bd-I ubiquinol oxidase subunit X [EC:7.1.1.7]	MIZ03_0290
K00425	cytochrome bd ubiquinol oxidase subunit I [EC:7.1.1.7]	MIZ03_0288
K00426	cytochrome bd ubiquinol oxidase subunit II [EC:7.1.1.7]	MIZ03_0289
**Cytochrome c oxidase, cbb3-type**		
K00404	cytochrome c oxidase cbb3-type subunit I [EC:7.1.1.9]	MIZ03_2283
K00405	cytochrome c oxidase cbb3-type subunit II	MIZ03_2284
K00406	cytochrome c oxidase cbb3-type subunit III	MIZ03_2286
K00407	cytochrome c oxidase cbb3-type subunit IV	MIZ03_2285
**F-type ATPase**		
K02108	F-type H+-transporting ATPase subunit a	MIZ03_4797, MIZ03_4811
K02109	F-type H+-transporting ATPase subunit b	MIZ03_4795, MIZ03_4809
K02110	F-type H+-transporting ATPase subunit c	MIZ03_4796, MIZ03_4810
K02111	F-type H+/Na+-transporting ATPase subunit alpha [EC:7.1.2.27.2.2.1]	MIZ03_4793, MIZ03_4808
K02112	F-type H+/Na+-transporting ATPase subunit beta [EC:7.1.2.2 7.2.2.1]	MIZ03_4791, MIZ03_4815
K02113	F-type H+-transporting ATPase subunit delta	MIZ03_4794
K02114	F-type H+-transporting ATPase subunit epsilon	MIZ03_4790, MIZ03_4814
K02115	F-type H+-transporting ATPase subunit gamma	MIZ03_4792, MIZ03_4807
**Cell motility**		
**Bacterial chemotaxis**		
K00575	chemotaxis protein methyltransferase CheR [EC:2.1.1.80]	MIZ03_0089, MIZ03_3765, MIZ03_4307
K02556	chemotaxis protein MotA	MIZ03_1266, MIZ03_3804
K02557	chemotaxis protein MotB	MIZ03_1267, MIZ03_3805, MIZ03_3941
K03406	methyl-accepting chemotaxis protein	MIZ03_0079, MIZ03_0084, MIZ03_0086, MIZ03_0088, MIZ03_0266, MIZ03_2476, MIZ03_2559, MIZ03_2560, MIZ03_4308, MIZ03_4310, MIZ03_4312, MIZ03_4313, MIZ03_4315, MIZ03_4316, MIZ03_4736
K03407	two-component system, chemotaxis family, sensor kinase CheA [EC:2.7.13.3]	MIZ03_0082, MIZ03_1546, MIZ03_3767, MIZ03_4317
K03408	purine-binding chemotaxis protein CheW	MIZ03_0085, MIZ03_0087, MIZ03_3597, MIZ03_3766, MIZ03_4309
K03410	chemotaxis protein CheC	MIZ03_1658
K03411	chemotaxis protein CheD [EC:3.5.1.44]	MIZ03_0090, MIZ03_3764, MIZ03_4306
K03412	two-component system, chemotaxis family, protein-glutamate methylesterase/glutaminase [EC:3.1.1.61 3.5.1.44]	MIZ03_0091, MIZ03_2924, MIZ03_3763, MIZ03_4305
K03413	two-component system, chemotaxis family, chemotaxis protein CheY	MIZ03_0080, MIZ03_1545, MIZ03_1657, MIZ03_2188, MIZ03_3768, MIZ03_3806, MIZ03_4319, MIZ03_4322, MIZ03_4380
K03414	chemotaxis protein CheZ	MIZ03_3807
K03415	two-component system, chemotaxis family, chemotaxis protein CheV	MIZ03_4314, MIZ03_4336
K03776	aerotaxis receptor	MIZ03_0261
K13924	two-component system, chemotaxis family, CheB/CheR fusion protein [EC:2.1.1.80 3.1.1.61]	MIZ03_2952, MIZ03_4343
**Flagellar assembly**		
K02386	flagellar basal body P-ring formation protein FlgA	MIZ03_3815
K02387	flagellar basal-body rod protein FlgB	MIZ03_3816
K02388	flagellar basal-body rod protein FlgC	MIZ03_3817
K02389	flagellar basal-body rod modification protein FlgD	MIZ03_3818
K02390	flagellar hook protein FlgE	MIZ03_3819
K02391	flagellar basal-body rod protein FlgF	MIZ03_3820
K02392	flagellar basal-body rod protein FlgG	MIZ03_3821
K02393	flagellar L-ring protein FlgH	MIZ03_3822
K02394	flagellar P-ring protein FlgI	MIZ03_3823
K02395	peptidoglycan hydrolase FlgJ	MIZ03_3824
K02396	flagellar hook-associated protein 1	MIZ03_3825
K02397	flagellar hook-associated protein 3 FlgL	MIZ03_3826
K02398	negative regulator of flagellin synthesis FlgM	MIZ03_3814
K02400	flagellar biosynthesis protein FlhA	MIZ03_3809
K02401	flagellar biosynthesis protein FlhB	MIZ03_3808
K02402	flagellar transcriptional activator FlhC	MIZ03_3803
K02403	flagellar transcriptional activator FlhD	MIZ03_3802
K02405	RNA polymerase sigma factor FliA	MIZ03_3812
K02406	flagellin	MIZ03_3789, MIZ03_3790, MIZ03_3792
K02407	flagellar hook-associated protein 2	MIZ03_3788
K02408	flagellar hook-basal body complex protein FliE	MIZ03_3785
K02409	flagellar M-ring protein FliF	MIZ03_3784
K02410	flagellar motor switch protein FliG	MIZ03_3783
K02411	flagellar assembly protein FliH	MIZ03_3782
K02412	flagellum-specific ATP synthase [EC:7.4.2.8]	MIZ03_3781
K02413	flagellar protein FliJ	MIZ03_3780
K02414	flagellar hook-length control protein FliK	MIZ03_3779
K02415	flagellar protein FliL	MIZ03_3778
K02416	flagellar motor switch protein FliM	MIZ03_3777
K02417	flagellar motor switch protein FliN	MIZ03_3776
K02418	flagellar protein FliO/FliZ	MIZ03_3775
K02419	flagellar biosynthesis protein FliP	MIZ03_3774
K02420	flagellar biosynthesis protein FliQ	MIZ03_3773
K02421	flagellar biosynthesis protein FliR	MIZ03_3772
K02422	flagellar secretion chaperone FliS	MIZ03_3787
K02423	flagellar protein FliT	MIZ03_3786
K02556	chemotaxis protein MotA	MIZ03_1266, MIZ03_3804
K02557	chemotaxis protein MotB	MIZ03_1267, MIZ03_3805, MIZ03_3941
K03086	RNA polymerase primary sigma factor	MIZ03_2821
K03092	RNA polymerase sigma-54 factor	MIZ03_1205

Table S5. List of CDSs for cytochrome c

Locus tag	Length (amino acids)	Number of CXXCH motifs	Annotation by FeGenie or blastp	Subcellular location predicted by Psortb	e-value
MIZ03_0014	206	2		Periplasmic	9.84
MIZ03_0044	116	1		Unknown	-
MIZ03_0045	197	4	CymA	CytoplasmicMembrane	9.82
MIZ03_0046	163	1		Periplasmic	9.84
MIZ03_0211	114	1		Unknown	-
MIZ03_0214	102	2		Unknown	-
MIZ03_0237	157	1		Unknown	-
MIZ03_0279	407	2		Periplasmic	10
MIZ03_0419	1109	1		CytoplasmicMembrane	9.82
MIZ03_0505	150	1		Periplasmic	10
MIZ03_0572	147	2		Cytoplasmic	9.26
MIZ03_0730	246	8		Cytoplasmic	8.96
MIZ03_0776	602	1		Unknown	-
MIZ03_0778	125	1		Cytoplasmic	8.96
MIZ03_0809	96	1		Unknown	-
MIZ03_0842	111	1		Cytoplasmic	8.96
MIZ03_0863	618	1		Cytoplasmic	9.97
MIZ03_0968	158	1		Periplasmic	9.84
MIZ03_1005	141	1		Periplasmic	9.76
MIZ03_1106	334	1		Cytoplasmic	8.96
MIZ03_1153	99	1		Periplasmic	9.44
MIZ03_1154	126	1		Periplasmic	10
MIZ03_1156	164	1		Unknown	-
MIZ03_1163	91	1		Unknown	-
MIZ03_1372	156	1		Unknown	-
MIZ03_1406	434	1		Cytoplasmic	9.97
MIZ03_1423	437	1		Periplasmic	9.84
MIZ03_1508	171	2		CytoplasmicMembrane	9.86
MIZ03_1614	114	1		Unknown	-
MIZ03_1615	842	10	MtrC/MtoC	Unknown	-
MIZ03_1616	345	10	MtrA/MtoA	Periplasmic	9.84
MIZ03_1618	662	8	DFE_0462	CytoplasmicMembrane	10
MIZ03_1636	185	1		Unknown	-
MIZ03_1752	112	1		Cytoplasmic	8.96
MIZ03_1760	507	1		CytoplasmicMembrane	9.82
MIZ03_1766	145	1		CytoplasmicMembrane	9.82
MIZ03_1878	330	10	MtrA/MtoA	Periplasmic	9.83
MIZ03_1880	213	2	Cyc1	Periplasmic	9.76
MIZ03_1881	218	2	Cyc1	Periplasmic	9.76
MIZ03_1882	148	2		Unknown	-
MIZ03_2035	378	2		Unknown	-
MIZ03_2069	217	1		Unknown	-

MIZ03_2070	266	1		Periplasmic	9.84
MIZ03_2073	366	2		Periplasmic	9.76
MIZ03_2075	105	1		Unknown	-
MIZ03_2080	199	2		Periplasmic	9.76
MIZ03_2082	100	1		Periplasmic	10
MIZ03_2115	652	1		Cytoplasmic	9.97
MIZ03_2116	165	3		Unknown	-
MIZ03_2125	242	1		Cytoplasmic	9.97
MIZ03_2141	181	1		Cytoplasmic	8.96
MIZ03_2271	100	1		Periplasmic	10
MIZ03_2284	208	1		Unknown	-
MIZ03_2286	299	2		Periplasmic	9.83
MIZ03_2437	615	9		Unknown	-
MIZ03_2608	334	1		Cytoplasmic	9.26
MIZ03_2728	107	1		Unknown	-
MIZ03_2739	511	8		Periplasmic	10
MIZ03_2756	184	1		Unknown	-
MIZ03_2787	196	1		Cytoplasmic	9.97
MIZ03_2833	534	8		Periplasmic	9.84
MIZ03_2842	196	1		Unknown	-
MIZ03_3038	522	1		CytoplasmicMembrane	7.88
MIZ03_3241	331	2	MacA	Periplasmic	10
MIZ03_3427	1309	1		Cytoplasmic	9.97
MIZ03_3441	716	1		Cytoplasmic	9.97
MIZ03_3530	229	1		CytoplasmicMembrane	9.86
MIZ03_3581	209	2		Periplasmic	10
MIZ03_3700	192	1		Cytoplasmic	9.97
MIZ03_3716	219	2		Periplasmic	10
MIZ03_3750	231	1		Cytoplasmic	9.26
MIZ03_3755	228	2		Periplasmic	9.76
MIZ03_3990	398	2		CytoplasmicMembrane	10
MIZ03_3991	160	2		Periplasmic	9.84
MIZ03_3992	129	1		Unknown	-
MIZ03_3993	152	2		Periplasmic	9.83
MIZ03_4042	229	1		Cytoplasmic	8.96
MIZ03_4090	411	1		CytoplasmicMembrane	10
MIZ03_4236	174	2		Unknown	-
MIZ03_4240	297	2	FoxE	Unknown	-
MIZ03_4341	157	1		Periplasmic	10
MIZ03_4385	323	1		Unknown	-
MIZ03_4542	130	1		Unknown	-
MIZ03_4644	618	1		CytoplasmicMembrane	10
MIZ03_4657	524	1		CytoplasmicMembrane	10
MIZ03_4691	59	1		Unknown	-
MIZ03_4697	181	1		Periplasmic	9.83
